# Sustained Attentional States Require Distinct Temporal Involvement of the Dorsal and Ventral Medial Prefrontal Cortex

**DOI:** 10.3389/fncir.2016.00070

**Published:** 2016-08-31

**Authors:** Antonio Luchicchi, Ouissame Mnie-Filali, Huub Terra, Bastiaan Bruinsma, Sybren F. de Kloet, Joshua Obermayer, Tim S. Heistek, Roel de Haan, Christiaan P. J. de Kock, Karl Deisseroth, Tommy Pattij, Huibert D. Mansvelder

**Affiliations:** ^1^Department of Integrative Neurophysiology, Center for Neurogenomics and Cognitive Research, VU University AmsterdamAmsterdam, Netherlands; ^2^Department of Bioengineering, Stanford UniversityStanford, CA, USA; ^3^Department of Anatomy and Neurosciences, VU University Medical CenterAmsterdam, Netherlands

**Keywords:** attention, dorsomedial prefrontal cortex, ventromedial prefrontal cortex, optogenetics, pyramidal neurons

## Abstract

Attending the sensory environment for cue detection is a cognitive operation that occurs on a time scale of seconds. The dorsal and ventral medial prefrontal cortex (mPFC) contribute to separate aspects of attentional processing. Pyramidal neurons in different parts of the mPFC are active during cognitive behavior, yet whether this activity is causally underlying attentional processing is not known. We aimed to determine the precise temporal requirements for activation of the mPFC subregions during the seconds prior to cue detection. To test this, we used optogenetic silencing of dorsal or ventral mPFC pyramidal neurons at defined time windows during a sustained attentional state. We find that the requirement of ventral mPFC pyramidal neuron activity is strictly time-locked to stimulus detection. Inhibiting the ventral mPFC 2 s before or during cue presentation reduces response accuracy and hampers behavioral inhibition. The requirement for dorsal mPFC activity on the other hand is temporally more loosely related to a preparatory attentional state, and short lapses in pyramidal neuron activity in dorsal mPFC do not affect performance. This only occurs when the dorsal mPFC is inhibited during the entire preparatory period. Together, our results reveal that a dissociable temporal recruitment of ventral and dorsal mPFC is required during attentional processing.

## Introduction

The medial prefrontal cortex (mPFC) plays a crucial role in several cognitive functions, among which attentional processes (Dalley et al., 2004). Pharmacological and lesion studies in rodents performing in different visual attention probing paradigms, including the 5-choice serial reaction time task (5-CSRTT) (Olton et al., 1988; Muir et al., 1996; Granon et al., 1998; Broersen and Uylings, 1999; Robbins, 2002; Kahn et al., 2012), have shown that deactivation of the mPFC impairs rodent performance (Muir et al., 1996). Furthermore, more detailed investigations have pointed toward a functional diversity in the management of various visuospatial attention-related functions by different mPFC areas (Passetti et al., 2002; Dalley et al., 2004). Along the dorsomedial-ventromedial axis of the PFC, the most dorsal subregions (including anterior cingulated cortex, ACg) might more prominently participate in sustained attentional states, controlling accuracy of responding to light cues as well as omission rates (Passetti et al., 2002; Dalley et al., 2004), whereas the ventral stations (prelimbic and infralimbic cortices) might be more involved in executive functions such as inhibition of inappropriate responses and behavioral flexibility (Chudasama and Muir, 2001; Passetti et al., 2002; Chudasama et al., 2003).

Pharmacological interventions and lesions of brain regions interfere with brain function on a time scale of hours to weeks, thereby exceeding the time scale of attentional processing. When an organism pays attention to its sensory environment for accurate detection of sensory cues in demanding tasks, attention-related neuronal activity typically occurs on a time scale of seconds (Totah et al., 2009, 2013; Donnelly et al., 2015; Kim et al., 2016). During these seconds of changed neuronal activity, both the ACg and the ventral regions of the mPFC process information to prepare the organism to respond to a stimulus (Totah et al., 2009). It was shown recently that activity of fast-spiking parvalbumin-containing interneurons in the mPFC is required for attentional processing, since optogenetic inhibition of these neurons on a seconds time-scale increases errors in performance (Kim et al., 2016). In addition, it has been reported that mPFC GABA interneurons might be crucially involved in the modulation of executive functions (Cho et al., 2015). Despite this, it is unknown how activity of pyramidal neurons in specific subcompartments of the mPFC is causally related to attentional processing in the seconds that precede the cue presentation as well as in the actual period of instrumental action, when rodents have to produce an adaptive response to the stimulus.

Pyramidal neurons represent 80–90% of cells in the mPFC (Riga et al., 2014) and their laminar organization renders their role in complex cognitive functions difficult to disentangle. For example, it has been shown that while superficial layer pyramidal neurons send their projections mainly intracortically, deep layer cells (among which those residing in layer V-VI) send efferent connection to subcortical and limbic structures (Douglas and Martin, 2004). Notably, layer V-VI cells in the mPFC are also strongly interconnected with the mediodorsal thalamus (Gabbott et al., 2005), a crucial region for the modulation of cognitive flexibility (Parnaudeau et al., 2015) and attention-related functions (Chudasama and Muir, 2001).

Due to the importance of pyramidal neurons in attentional processing, we addressed here the temporal requirements for activation of pyramidal neurons in the dorsomedial PFC (DmPFC, encompassing the ACg and the dorsal portion of the PL) and ventromedial PFC (VmPFC, centered in the border between the ventral part of PL and the dorsal IL) in rats performing in the 5-CSRTT. Since attention is a multi-dimensional construct, this task assesses aspects of a sustained visuospatial attentive state by testing the ability to monitor 5 different spatial locations over an extensive amount of trials. In addition, the task also provides information on other behavioral functions such as motivation, motor behavior, inhibitory control, decision-making strategies and timing (see for review Robbins, 2002). Using the 5-CRSTT, we tested whether the involvement of DmPFC and VmPFC excitatory cells was required during specific phases of preparatory attentional states, or whether these two subcompartments modulate this function at different time-scales and epochs. By optogenetic silencing of either DmPFC or VmPFC pyramidal neurons (Yizhar et al., 2011) at defined time windows of a few seconds prior and during cue detection, we find that pyramidal neuron activity in DmPFC and VmPFC shows distinct temporal requirements during early and late phases of preparatory sustained attentional states, and during cue detection/instrumental action. These findings help to better disentangle the intricate network activity of the mPFC during complex cognitive tasks, providing a temporal view on mPFC activity requirements for adaptive and maladaptive behaviors.

## Materials and methods

### Animals

All experimental procedures were in accordance with European and Dutch law and approved by the animal ethical care committee of the VU University and VU University Medical Center. Male Long Evans wild-type rats (Janvier Labs, France; 8–10 weeks old at the start of the experiments) were used for all the experiments. Rats were individually housed on a 12 h light/dark reversed cycle (lights OFF: 7 a.m.). Only when assigned to behavioral experiments rats were food deprived. Food restriction began 1 week before the initiation of operant training in order to achieve and maintain about 85–90% of the free-feeding body weight. Water was provided *ad libitum*. In total 31 rats were included in this study (29 for behavioral testing and 2 for structural imaging).

### Opsin virus delivery and implantation of optic fibers

CaMKIIα promoter-driven opsin pAAV-enhanced halorhodopsin (eNPHR3.0)::eYFP, pAAV-enhanced archaerhodopsin (eARCH3.0)::eYFP and pAAV::eYFP were packaged as AAV serotype 2 virus (titer 1.0–6.0 × 10^12^). Rats were anesthetized with isoflurane (2.5%) and then mounted in a stereotaxic frame (Kopf instruments, Tujunga, USA). The skin of the scalp was retracted and 2 holes were drilled at the level of the medial prefrontal cortex (mPFC). Stainless steel micro-needles connected to a syringe (Hamilton, USA) were inserted at the desired coordinates to deliver the virus in the brain. For the DmPFC group, injections were made at AP +2.76 mm; ML ±1.49 mm; DV −2.94 and −2.84 mm from bregma (infusion angle 10°), while for the VmPFC group at AP +2.76 mm; ML ±1.45 mm; DV −4.87 and −4.77 mm from skull (10° infusion angle) (Paxinos and Watson, 2007). One microliter virus was injected per hemisphere in two steps of 500 nL at an infusion rate of 6 μL/h. A total of 8 rats were injected with AAV2-eNPhR3.0::EYFP, 13 with AAV2-eARCH3.0::EYFP and 8 with AAV2::EYFP. 14 rats in total were injected in the DmPFC and 15 rats were injected in the VmPFC (including control rats).

Then, 2 guide screws and 2 chronic implantable glass fibers (200 μm diameter, 0.20 numerical aperture, ThorLabs, Newton, NJ, USA) mounted in a sleeve (1.25 mm diameter; ThorLabs, Newton, NJ, USA) were placed in the rat brain. The fibers were implanted right on top of the viral injection location (200–300 μm on average). Finally, a double component dental cement (Pulpdent©, Watertown, USA) mixed with black carbon powder (Sigma Aldrich, USA) was used in order to secure the optic fibers. All the surgical manipulations were performed before the behavioral training and testing.

### Behavioral procedures

After 1 week of recovery from surgery and 1 week of habituation in the reverted light/dark cycle, rats started training in the 5-CSRTT in operant cages (Med Associates Inc., St. Albans, VT, USA). Training consisted of a period during which rats learned to respond to a brief visual cue that was randomly lit in one out of the five apertures of the operant cage (Bari et al., 2008). To associate cue with the delivery of reward rats were first trained with all the apertures illuminated (all holes on, **Figure 3B**) in order to learn that a nose-poke returns a food pellet and subsequently with only one aperture constantly illuminated (one hole on, **Figure 3B**) to learn responding into this illuminated aperture is associated with reward delivery. After the learning phase, titration of shortening the stimulus duration was based on individual performance of each rat, and was reduced from 16 to 1 s. Criteria to move to a shortened stimulus duration were the percentage of accuracy (>80%) and omitted trials (< 20%). Finally, when rats met the criteria at 1 s stimulus duration they were moved to the pretesting phase. In the pretesting phase, a green custom-made LED replaced the normal house-light of the operant cages, (< 1 mW intensity) to mask reflections by the laser light used for the experiments. The LED house-light did not affect performance when compared to normal house-light.

After three consecutive sessions during which rats performed according to the aforementioned criteria with the LED on, additional baseline sessions were conducted (3 consecutive sessions). During these sessions subjects were connected to the patch-cable (Doric Lenses, Quebec city, Canada) used to deliver the light into the brain. In this condition, accuracy was typically above 80%. However, they often did not show less than 20% omissions. This was most likely due to the fact that the animals were connected to the optic fiber patch cable and therefore less free to move in combination with the short time window for the animal to respond (i.e., within 2 s after the cue light went off). This parameter makes the paradigm more demanding than other versions of the 5-CSRTT in which response time is usually set to 5 s (Passetti et al., 2002). Therefore, the omission criterion was increased to less than 40% omissions.

After acquisition of baseline rats were assigned to the testing phase where the task comprised 100 consecutive trials with a random assignment to the condition of laser ON or laser OFF (see below). In the whole text we refer to completed trials (correct, incorrect, omissions) while in the 100 trials premature responses are left apart from the count.

To light-activate the opsins *in vivo*, we used a diode-pumped laser (532 nm, Shanghai Laser and Optics Century Co, China) directly connected to the rat optic glass fiber implant. Light was delivered at 9–12 mW for experiments performed with eNPhR3.0 and at 7–8 mW for experiments carried out with eARCH3.0. These stimulation regimens are able to produce a theoretical irradiance which ranges between 9.76 and 13.01 mW/mm^2^ 500 μm from the fiber tip for the eNPhR3.0 experiments (corresponding to the center of the viral transfection) and ranging between 7.59 and 8.68 mW/mm^2^ for eARCH3.0 experiments (http://web.stanford.edu/group/dlab/cgi-bin/graph/chart.php).

Light was delivered according to scheduled epochs by a stimulator (master 9, AMPI Jerusalem, Israel) connected to the computer interface.

For the testing phase, the following parameters have been acquired and analyzed through a box-computer interface (Med-PC, USA) and custom written MATLAB scripts (Mathworks): *accuracy on responding to cues* (ratio between the number of correct responses per session over the sum between correct and incorrect hits, expressed as percentage); *absolute and percentage of correct, incorrect responses and errors of omission; correct or incorrect response latency; latency to collect reward; number of premature and perseverative responses*. Percent of correct, incorrect and omissions were calculated based on the number of started trials (Semenova et al., 2007).

In line with previous studies (Pinto et al., 2013), no differences were found in behavioral effects of eARCH3.0 and eNPhR3.0 injected animals (data not shown). Therefore, data from eARCH3.0 and eNPhR3.0 injected animals were pooled.

### Optical inhibition protocols

Rats were randomly assigned to different stimulation protocols and received different optical inhibition epochs. Optical inhibition sessions were done 2–3 times a week with a baseline session in between to control for potential carry-over effects. Rats were tested according to the following optical inhibition protocols: (a) 3 s at the trial onset, (b) 2 s at the end of the preparatory period of a sustained attentional state, (c) 5 s throughout the whole preparatory period, (d) 1 s during light cue presentation. During a session, animals received only one light stimulation protocol. We chose these light regimens to make a clear distinction between prestimulus period and stimulus presentation/instrumental response period (Totah et al., 2013) (protocol a, b, and c vs. protocol d) and to differentiate between the whole pre-cue period and the period which consists in the actual orienting activity of the rat toward the task ports (Totah et al., 2009, 2013; Donnelly et al., 2015) (protocol c vs. protocol b). Light-ON and light-OFF trials were assigned semi-randomly with approximately 50% ON trials and 50% OFF trials. The majority of animals (28 out of 29) completed 100 trials within the first 20–25 min. One animal did not complete 100 trials before the time cut off of 60 min. Whereas animals were tested in all four different optical inhibition protocols, in some rats due to fiber loss not all protocols could be completed. Moreover, reported data for the majority of rats refer to the first optical inhibition session after establishment of stable baseline performance. In some cases, as described below, rats were retested in the same optical inhibition session.

### Exclusion criteria

Single sessions were excluded from analysis when technical problems (i.e., patch-cables disconnected during the task) made the results unreliable. In all these cases, we repeated the same protocol after re-acquisition of baseline criteria and used data from these sessions.

### Histological verification

After behavioral testing, brains were checked for fiber placement and viral expression. For this, rats were anesthetized with isoflurane and a mix of ketamine (200 mg/kg i.p.) and dormitol (100 mg/kg i.p.) and then transcardially perfused (50–100 mL NaCl and 200–400 mL PFA 4%). Brains were removed and maintained in 4% PFA for at least 24 h. After that, brains were sliced with a vibratome (Leica Biosystem, Germany) into 50–100 μm coronal sections and mPFC slices were mounted on glass slides covered by 2% Mowiol and anti-fading mounting covers. Images were taken with a confocal microscope (LSM 510 Meta; Zeiss, Germany) with excitation wavelength of 514 nm bandpass filtered between 530 and 600 nm, and further analyzed using ImageJ (NIH, USA).

### *In vitro* physiological recordings

Following behavioral testing, five rats (by that time 8–10 months old) were used for electrophysiological recordings. Animals were anesthetized with 5% isoflurane and an i.p. injection of 0.1 ml/g Pentobarbital and subsequently perfused with 35 ml of ice-cold N-Methyl-D-glucamin solution (NMDG solution; in mM: NMDG 93, KCl 2.5, NaH2PO4 1.2, NaHCO3 30, HEPES 20, Glucose 25, NAC 12, Sodium ascorbate 5, Sodium pyruvate 3, MgSO410, CaCl2 0.5, at pH 7.4 adjusted with 10M HCl). After decapitation the brain was removed and incubated for 10 min in ice-cold NMDG solution. Coronal mPFC slices (350 μm) were made in ice-cold NMDG solution and incubated afterwards for 3 min in 34°C NMDG solution. Slices were maintained in an incubation chamber for at least 1 h before recordings were conducted at room temperature in oxygenated holding solution containing the following (Holding solution; in mM): NaCl 92, KCl 2.5, NaH2PO4 1.2, NaHCO3 30, HEPES 20, Glucose 25, NAC 1, Sodium ascorbate 5, Sodium pyruvate 3, MgSO4 0.5, CaCl2 1M.

Whole-cell recordings from pyramidal neurons were made at 32°C in oxygenated artificial cerebrospinal fluid (ACSF; in mM: NaCl 125, KCl 3, NaH2PO4 1.25,MgSO4 1, CaCl2 2, NaHCO3 26, Glucose 10). For recordings a potassium-based internal solution was used (in mM: K-gluconate 135, NaCl 4, Hepes 10, Mg-ATP 2,K2Phos 10, GTP 0.3, EGTA 0.2) with patch-pipettes that had a resistance of 3–6 MΩ. Recorded neurons were kept at a holding potential close to −70 mV.

For recordings Multiclamp 700/B amplifiers (Molecular Devices) were used and data was collected with a sampling rate of 10 kHz and low-pass filtering at 3 kHz (Axon Digidata 1440A and pClamp 10 software; Molecular Devices).

### Optogenetic slice stimulation

To optically activate opsins, green light (530 nm) was applied to the slices. Light pulses were evoked by using a DC4100 4-channel LED-driver (Thorlabs, Newton, NJ) or a Fluorescence lamp (X-Cite Series 120q, Lumen Dynamics). During recordings fifty sweeps, each 10 s apart were applied. One sweep consists of a single light pulse with a duration of 1 or 5 s. These pulse regimes represent the shortest and the longest stimulation protocol used for behavioral experiments, respectively. The intensity of the light source was adjusted to 1.7, 3, 7, or 17 mW. For recording the in/output curves 1 s light pulse with all different stimulation intensities were applied for five sweeps with an interval of 10 s.

### Statistical analyses for behavioral experiments

To evaluate the main behavioral data between the opsin group and eYFP control group, two-way ANOVAs for repeated measures were performed. Corrected values for multiple comparison with Sidak's test were used when interaction between light and virus was significant. In all cases, the ANOVAs were preceded by the Kolmogorov-Smirnov (KS) test for normal distribution. In cases when the KS *p*-value was >0.05, factorial analysis was performed on the raw data per parameter. In the other cases, raw data were first transformed with square-root or arcsin transformation.

Data were analyzed by MATLAB 2014a (Mathworks), Microsoft Excel (Office) and graphs were plotted by GraphPad Prism. In all cases the significance level was *p* < 0.05.

## Results

**Figure 1:**
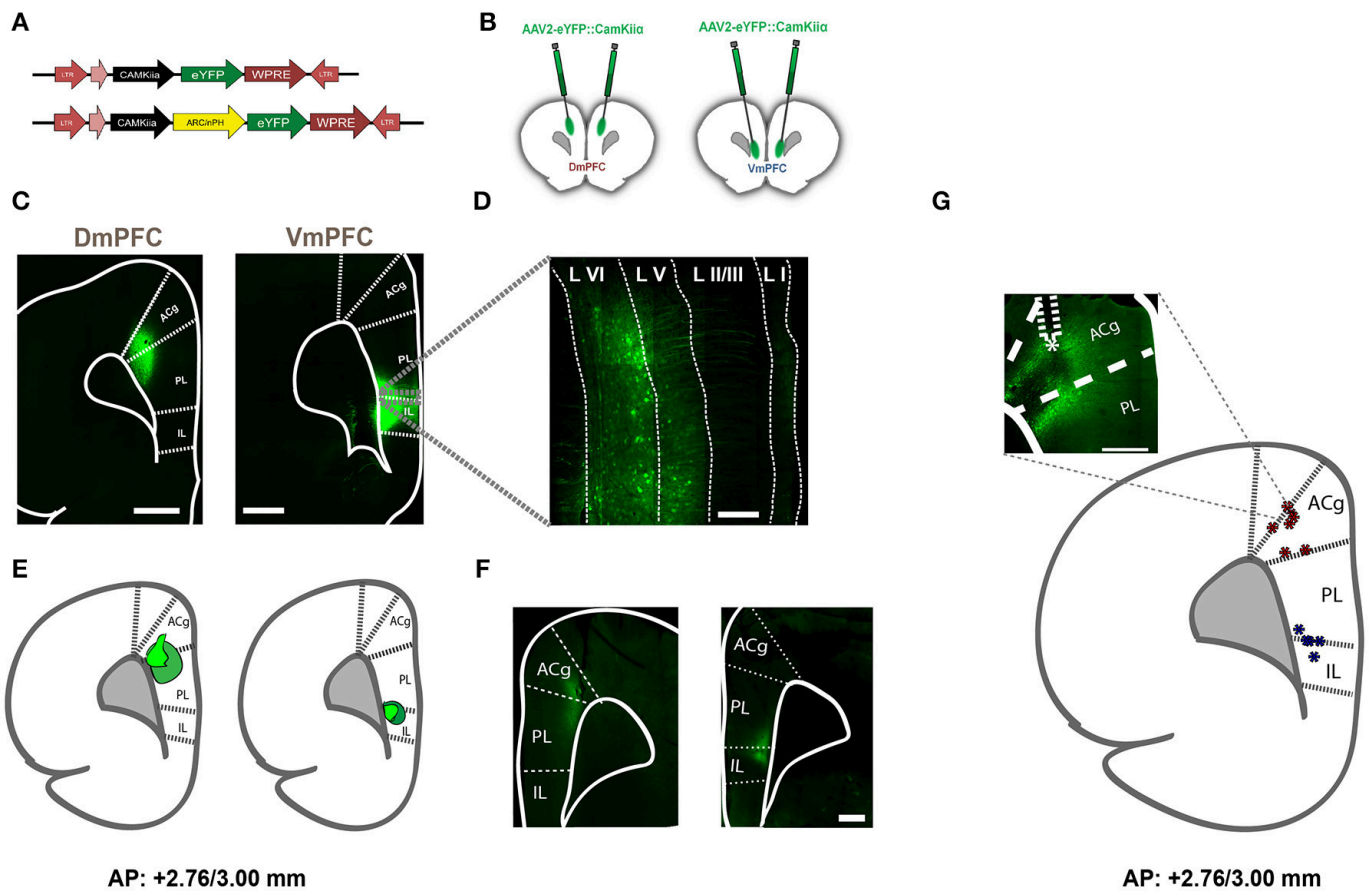
**Viral expression in rats injected with AAV2-eYFP, AAV2-eNPHR3.0, and AAV2-eARCH3.0 and optical fiber location to achieve selective illumination of either Dm- or VmPFC. (A)** Schematic representation of the viruses used to achieve expression of inhibitory opsins and eYFP in either DmPFC or VmPFC. **(B)** Graphic representation of the injections made in either the DmPFC or the VmPFC to test the spread of transfection of the virus in both regions **(C)** Overview (zoom 10×) of injection location in both the DmPFC (left panel) and the VmPFC (right panel). In this figure animals were injected with AAV2-eYFP::CamkIIα. Scale bar is 1 mm for both pictures. **(D)** Magnified (zoom 40×) confocal picture reporting an example of the transfected neurons by using the same viral plasmid used for the behavioral experiments. White dotted lines illustrate the empirical differentiation between the different mPFC layers, indicating that the majority of the transfected cells were in the deep-layers with a reduced amount in the upper-layers. Scale bar is 200 μm. Also in this example viral infusions were made using AAV2-eYFP::CamkIIα. **(E)** Visual identification of the virus spread in a sample of rats previously used to perform behavioral experiments and injected with either AAV2-eNPHR3.0-eYFP::CamkIIα or AAV2-eARCH3.0-eYFP::CamkIIα. Dark green wider circles represent the maximal expression achieved, while light green small shapes report the smallest expression detected (*n* = 10 in total). Confocal pictures of exemplificative images in this batch are reported in **(F)** (scale bar is 500 μm for both images). In this examples rats were injected with AAV2-eNPHR3.0-eYFP::CamkIIα. **(G)** Visual identification of fiber placement in a sample of rats previously used for 5-CSRTT experiments and injected with either AAV2-eNPHR3.0-eYFP:: CamkIIα or AAV2-eARCH3.0-eYFP::CamkIIα. Inset reports an example of the fiber location in the mPFC (scale bar is 500 μm) in a rat injected with AAV2-eARCH3.0-eYFP::CamkIIα. Blue asterisks are referred to optic fibers located to achieve regional inhibition in the VmPFC, while red asterisks report the same fiber placement in the DmPFC (*n* = 12 in total).

**Figure 2:**
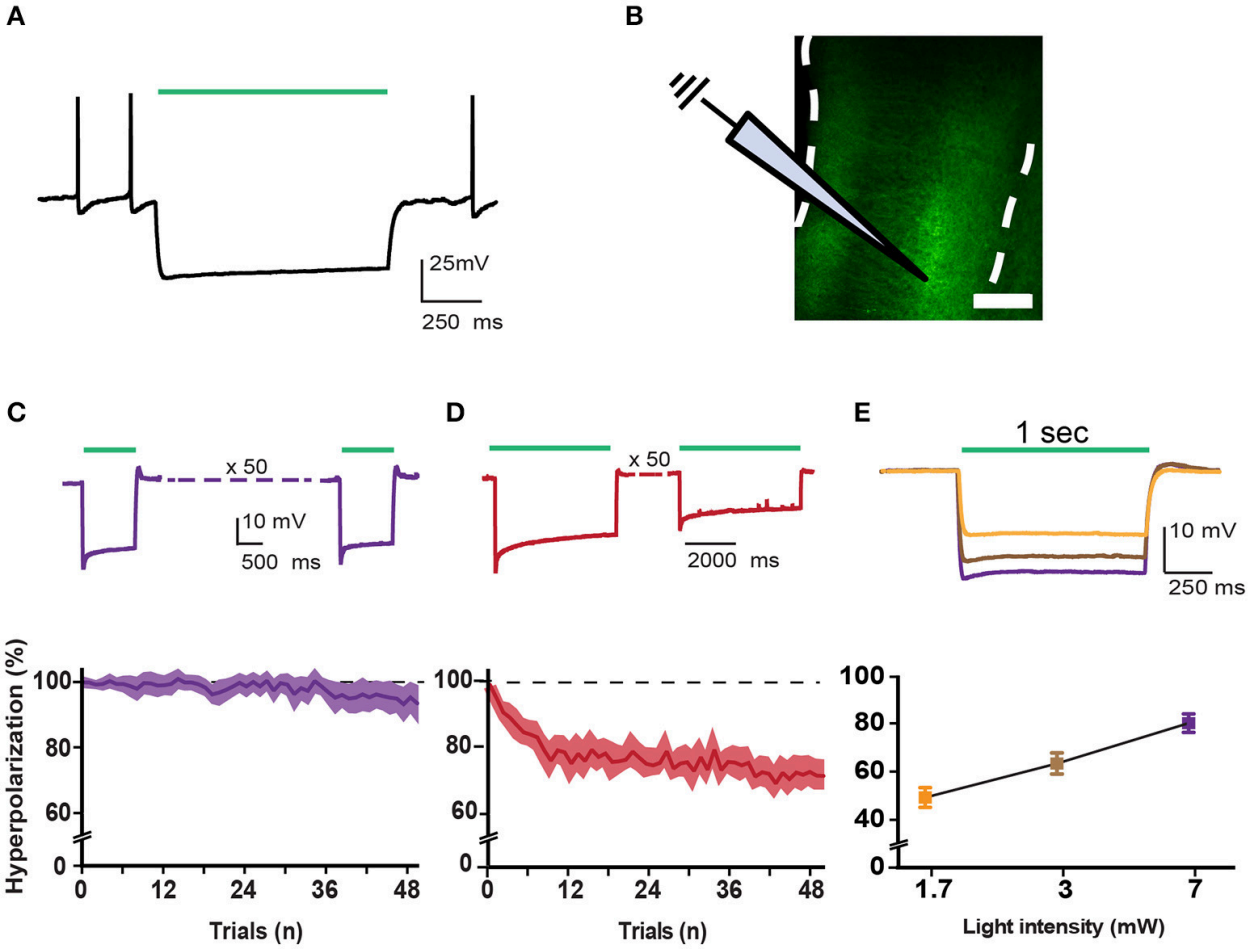
**Correct incorporation of inhibitory opsins in pyramidal cells. (A)** Trace showing a typical eARCH3.0-mediated voltage waveform in a layer V pyramidal neuron in response to green light (530 nm, 1 s, 7 mW). **(B)** Schematic representation of recording configuration in mPFC coronal slices of a rat. White dotted lines represent the borders of the mPFC. Scale bar is 200 μm. **(C)** Top panel shows characteristic voltage waveforms monitored in response to one green light pulse (1 s duration: *n* = 14) in a layer VI pyramidal neuron transfected with the AAV2-eARCH3.0::eYFP. Bottom panel graph reports the normalized hyperpolarization amplitude of each trial (50 trials, 1 s light pulse, repeated each 10 s, 7 mW light intensity). All responses were normalized to the maximal amplitude of the first response (graph report values as mean ± S.E.M.). **(D)** top and bottom panels report the same example and analysis showed in **(C)** with a longer light pulse (5 s; *n* = 13). **(E)** Example traces show that pyramidal neurons responded to light pulses in an intensity-dependent fashion, with more pronounced hyperpolarization following higher light intensities (top panel). Bottom panel shows an input/output curve for different light intensities (*n* = 11 neurons, data are reported as mean ± S.E.M.). Percentage of hyperpolarization: 1.7 mW = 49.28 ± 4.09%; 3 mW = 63.39 ± 4.377%; 7 mW = 80.11 ± 3.812%, Data are normalized in each cell to the maximal response (evoked by a 17 mW light pulse). Average amplitude at 17 mW light pulses is −23.464 ± 3.361 mV (*n* = 22; data are reported as mean ± S.E.M.).

To express inhibitory opsins in excitatory pyramidal neurons of either DmPFC or VmPFC, we used an AAV2 plasmid containing the CamkIIα promoter driving expression of either archaerhodopsin (eARCH3.0) or halorhodopsin (eNPHR3.0) and eYFP (Yizhar et al., 2011). For the control group we injected the same virus with eYFP only (Figure 1A). Injections in the DmPFC targeted the border between the ventral part of the pregenual anterior cingulated cortex (ACg) and the dorsal part of the prelimbic cortex (PL), whereas VmPFC viral infusions transfected neurons in the ventral PL and the dorsal infralimbic cortex (IL) (Figures 1B,C). In both cases AAV2 injections primarily targeted the deep layers (layer V-VI) of the mPFC (Figure 1D). Same pattern was revealed in rats dissected after 5-CSRTT experiments (Figures 1E,F), where also fiber placement in both the Dm- and the VmPFC was mainly located in the area ranging from layer V to layer VI (Figure 1G). Whole-cell patch clamp recordings performed in rats that previously were tested in the 5-CSRTT, confirmed the correct expression of the inhibitory opsins eNPHr3.0 or eARCH3.0 in pyramidal cells. Brief light pulses of similar length as used for the behavioral experiments (1 or 5 s; 530 nm) triggered after 50 consecutive repetitions a marked hyperpolarization response in the recorded cells (Figures 2A–D). Hyperpolarization remained stable across the different trials (Figures 2C,D), with a slight reduction (about 20%) when light was consecutively delivered at the duration of 5 s (Figure 2D). In addition, input/output curves confirmed that: (a) light manipulation of pyramidal neurons was intensity-dependent, with stronger hyperpolarization following higher light intensity and that (b) also the lowest light intensity (1.3 mW) produced a sustained hyperpolarization of the cells (Figure 2E). We did not observe rebound action potentials following light-induced inhibition.

### Transient mPFC inhibition immediately before and during cue presentation

**Figure 3:**
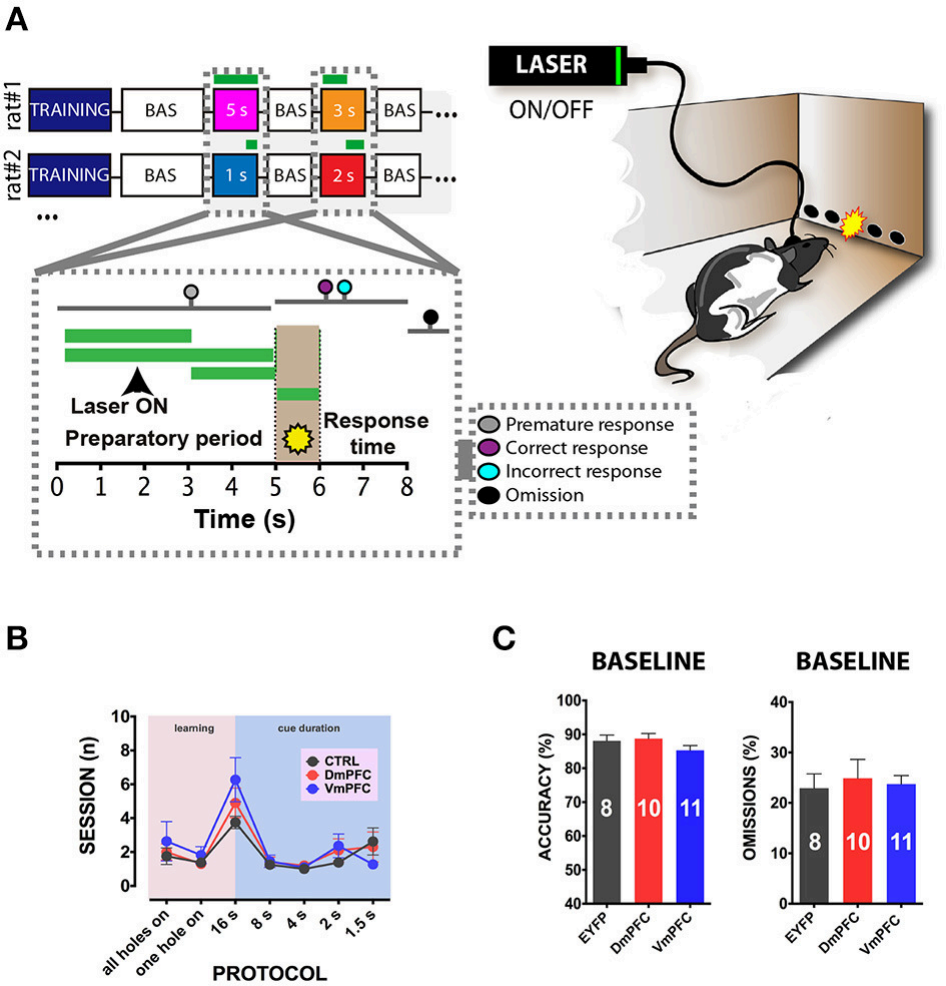
**5-CSRTT: protocols, training and baseline performance. (A)** After stable baseline performance (BAS) for three consecutive sessions rats were assigned to the testing phase. Colored squares in the top-right panel represent the different light epochs of stimulation used. Numbers represent the length of the stimulation per session. White squares in between the stimulation days represent a baseline session when no light was delivered in the brain. Bottom-right panel represents a schematic picture of a single trial of the task. The first 5 s reported in the x axis shows the preparatory period of sustained attentional state, the light brown period (5th to 6th s in the x axis) refers to the presentation of the cue, and the last 2 s represent the limited hold period. Colored dots represent the possible responses that were recorded during the session. Responses before cue presentation were considered as premature and punished with a 5 s time-out period. Correct responses were rewarded with a food pellet, whereas incorrect pokes were punished with a time-out period. If a response did not occur within the limited hold period, an omitted trial was recorded. Green lines represent the different light epochs (see methods). Left panel reports a representative illustration of a rat performing in the 5-CSRTT. Rats are bilaterally connected via patch cables to a laser, which delivers (ON) or does not deliver (OFF) light in the desired epoch. The percentage of trials with light ON and OFF was approximately fifty for both options. **(B)** Illustration of the number of sessions within each training phase and stimulus duration of the task for the three different groups of rats included in the study (CTRL: *n* = 8; DmPFC: *n* = 10; VmPFC: *n* = 11; data are expressed as mean ± S.E.M.). **(C)** Graphs illustrating the averaged baseline with cables in accuracy and omissions for the 3 groups. Results are expressed as mean ± S.E.M.

**Figure 4:**
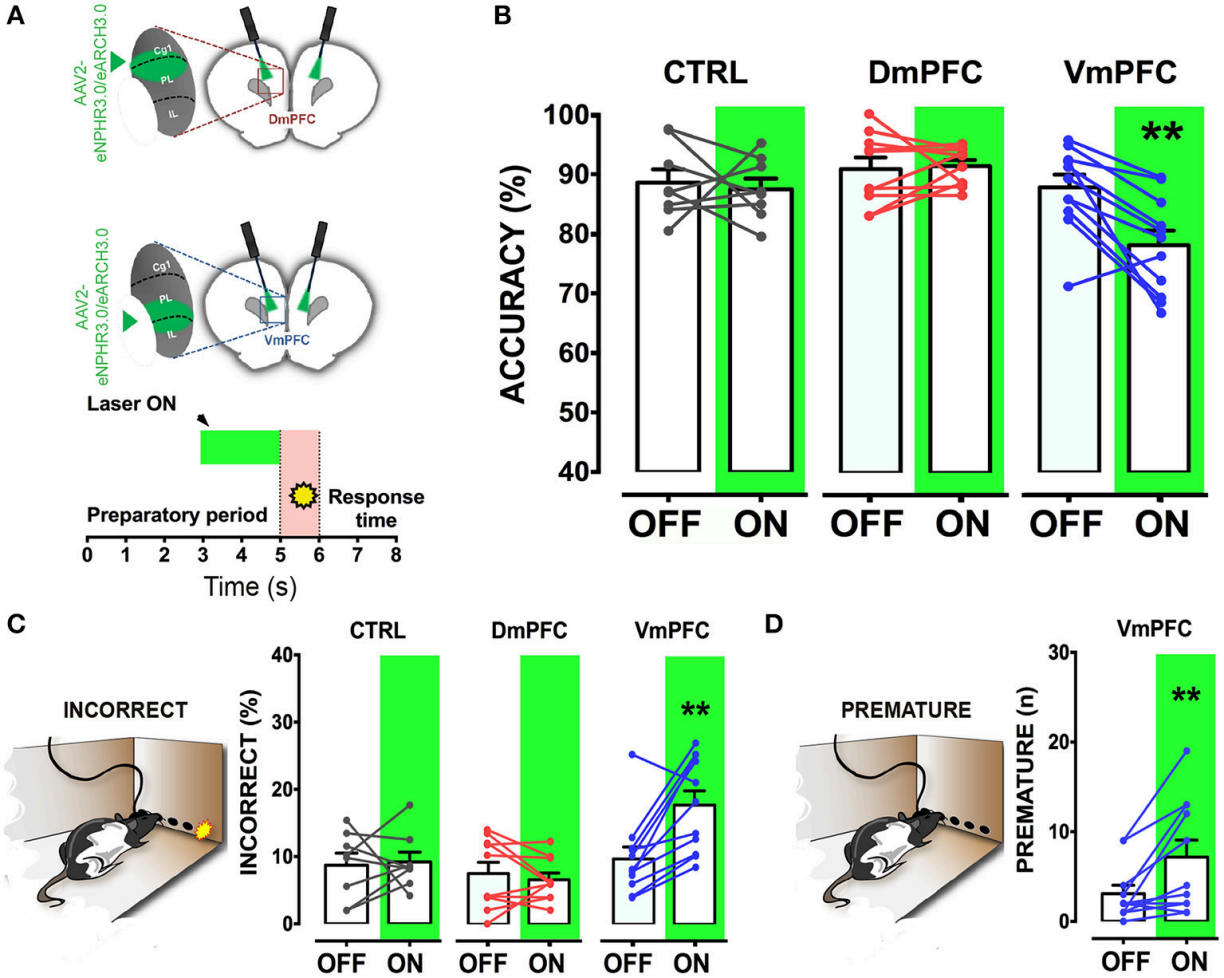
**VmPFC inhibition affects sustained attentional state seconds before cue presentation. (A)** Top panel shows a schematic representation of the optogenetic inhibition of either the DmPFC or the VmPFC. Optic fibers were placed 200–300 μm above the viral infusion location. Insets represent the target area in the two subregions. Bottom panel shows a graphical representation of the light protocol used to achieve the mPFC inhibition 2 s before cue presentation. **(B)** Accuracy of performance in controls (CTRL; *n* = 8), DmPFC (*n* = 10), and VmPFC (*n* = 11) injected animals **(C)** Percent of incorrect responses and **(D)** number of premature responses in the different groups. Asterisks indicate the result of the *post-hoc* multiple comparison Sidak's test. ^**^p < 0.01. All numbers and statistical results are available in Table 1.

To address whether a reversible inactivation of pyramidal neuron activity in either Dm or VmPFC affects rodent performance at specific time points during a preparatory attentional state, we trained rats in the 5-CSRTT (Figure 3A) and tested the effect of subregion-specific deactivation during precise time-windows in the task (see methods). Neither training [two-way ANOVA, effect of interaction group x protocol: *F*_(12, 156)_ = 0.992; *p* = 0.452; effect of group: *F*_(2, 26)_ = 0.684; *p* = 0.513; Figure 3B], nor baseline performance differed between groups [Accuracy: one-way ANOVA: *F*_(2, 28)_ = 1.607; *p* = 0.220; omissions: one-way ANOVA: *F*_(2, 28)_ = 0.117; *p* = 0.893; Figure 3C]. During the preparatory period, when the animal is actively attending the cue-holes, single-units in the ACg and PL area show a transient pre-cue increase in firing rate (Totah et al., 2009). However, it is not known whether this activity causally drives a sustained attentional state. To test whether increased activity during this period in either DmPFC or VmPFC is required for proper performance, pyramidal neurons in either of these subregions were inhibited by light for 2 s prior to cue presentation (Figure 4A), during the time window that represents the actual period when the rat orients and actively awaits the upcoming stimulus, before it is required to produce a response to the cue (Totah et al., 2013). Only inhibition of VmPFC pyramidal neurons resulted in a reduction of accuracy of responding [two-way repeated measures ANOVA: effect of light x virus interaction: *F*_(2, 26)_ = 5.984; *p* = 0.007; effect of virus: *F*_(2, 26)_ = 6.154; *p* = 0.006; effect of light: *F*_(1, 26)_ = 4.175; *p* = 0.051; Sidak's multiple comparison test OFF vs. ON: CTRL: *p* = 0.965; DmPFC: *p* = 0.854; VmPFC: *p* = 0.001; Figure 4B]. This effect was primarily due to an increase in the percentage of incorrect responses [two-way repeated measures ANOVA: effect of light x virus interaction: *F*_(2, 26)_ = 4.115; *p* = 0.028; Sidak's multiple comparison test OFF vs. ON: CTRL: *p* = 0.952; DmPFC: *p* = 0.999; VmPFC: *p* = 0.002; Figure 4C], and accompanied by an increase in premature responses (Wilcoxon matched-pairs signed rank test; *p* = 0.008; Figure 4D). Inhibition of pyramidal neurons in the DmPFC 2 s prior to cue presentation did not affect any parameter of performance in the 5-CSRTT (Figure 4B, Table 1). These results suggest that a reduction in accurate responding might be due to the reduced ability to control inappropriate responses when VmPFC activity is inhibited for 2 s before cue presentation.

**Table 1:** **Complete overview of the different parameters analyzed in the 5CSRTT under the four different light epochs**.

	**CTRL**		**DmPFC**		**VmPFC**	
**ACCURACY (%)**	**OFF**	**ON**	**OFF**	**ON**	**OFF**	**ON**
2 s before cue 1 s during cue First 3 s of the trial 5 s before cue	88.78 ± 2.21 87.54 ± 3.79 86.88 ± 3.02 85.39 ± 3.04	87.61 ± 1.83 82.21 ± 5.14 84.24 ± 4.79 88.58 ± 3.81	90.69 ± 1.91 86.41 ± 2.04 88.55 ± 2.19 93.21 ± 1.65	91.18 ± 1.00 90.19 ± 2.39 89.38 ± 2.12 88.26 ± 2.41^*^	87.6 ± 2.11 87.99 ± 2.10 89.68 ± 1.64 85.76 ± 1.19	77.93 ± 2.44^*^ 79.09 ± 3.85^*^ 85.23 ± 3.43 90.01 ± 1.38^*^
**OMISSIONS (%)**						
2 s before cue 1 s during cue First 3 s of the trial 5 s before cue	22.07 ± 2.98 15.69 ± 2.19 14.19 ± 1.04 19.65 ± 4.99	23.45 ± 5.55 15.13 ± 2.86 12.37 ± 1.7 22.56 ± 6.43	21.37 ± 2.91 18.4 ± 3.66 13.3 ± 3.15 24.67 ± 3.95	27.35 ± 5.14 16.7 ± 7.45 19.72 ± 5.44 29.45 ± 4.82	21.32 ± 2.7 13.2 ± 2.73 11.75 ± 1.1 15.84 ± 2.79	19.85 ± 3.91 15.5 ± 4.61 14.54 ± 1.02 16.35 ± 3.27
**CORRECT (%)**						
2 s before cue 1 s during cue First 3 s of the trial 5 s before cue	69.18 ± 3.16 73.50 ± 3.87 74.63 ± 3.33 68.54 ± 4.91	67.34 ± 5.54 69.74 ± 4.49 73.74 ± 4.19 68.08 ± 5.86	71.07 ± 2.37 76.53 ± 2.40 76.34 ± 2.51 69.93 ± 3.36	66.23 ± 4.55 71.92 ± 5.22 72.13 ± 5.22 61.79 ± 3.58^*^	69.00 ± 3.16 76.29 ± 2.53 79.21 ± 2.28 72.05 ± 2.00	62.52 ± 3.94 66.76 ± 4.55^*^ 72.99 ± 3.72 75.27 ± 2.32
**INCORRECT (%)**						
2 s before cue 1 s during cue First 3 s of the trial 5 s before cue	8.74 ± 1.79 10.4 ± 3.04 11.18 ± 2.5 11.81 ± 2.73	9.21 ± 1.47 15.21 ± 4.38 13.83 ± 4.12 9.37 ± 3.16	7.55 ± 1.65 10.16 ± 2.19 10.11 ± 2.16 5.39 ± 1.38	6.62 ± 1.00 8.36 ± 1.63 8.39 ± 1.64 8.95 ± 2.29^*^	9.66 ± 1.76 10.51 ± 1.93 9.04 ± 1.32 12.11 ± 1.28	17.62 ± 2.1^*^ 17.73 ± 3.59^*^ 12.46 ± 2.78 8.63 ± 1.41^*^
**PREMATURE (n)**						
2 s before cue 1 s during cue First 3 s of the trial 5 s before cue	3.37 ± 1.12 6.6 ± 2.2 4.75 ± 2.01 4.57 ± 1.7	5.62 ± 1.67 7 ± 2.53 3.5 ± 1.94 4.28 ± 1.64	2.2 ± 0.42 3.67 ± 0.67 2.57 ± 0.89 3.4 ± 1.27	2.8 ± 0.63 4.67 ± 1.93 2.00 ± 0.95 3.6 ± 1.45	3.09 ± 0.94 5.16 ± 3.00 4.17 ± 1.35 3.37 ± 0.96	7.18 ± 1.89^*^ 5.67 ± 1.43 4.5 ± 0.92 5.12 ± 1.27
**RESPONSE TIME CORRECT (sec)**						
2 s before cue 1 s during cue First 3 s of the trial 5 s before cue	0.68 ± 0.04 0.62 ± 0.03 0.63 ± 0.04 0.66 ± 0.05	0.69 ± 0.04 0.66 ± 0.01 0.65 ± 0.07 0.67 ± 0.05	0.71 ± 0.05 0.66 ± 0.04 0.64 ± 0.04 0.69 ± 0.04	0.69 ± 0.05 0.64 ± 0.04 0.64 ± 0.02 0.72 ± 0.06	0.78 ± 0.07 0.62 ± 0.03 0.61 ± 0.06 0.62 ± 0.04	0.87 ± 0.15 0.66 ± 0.05 0.61 ± 0.05 0.61 ± 0.04
**RESPONSE TIME INCORRECT (sec)**						
2 s before cue 1 s during cue First 3 s of the trial 5 s before cue	1.03 ± 0.18 0.81 ± 0.13 1.12 ± 0.09 1.00 ± 0.24	1.13 ± 0.12 1.04 ± 0.1 1.44 ± 0.29 0.96 ± 0.14	1.38 ± 0.18 0.91 ± 0.20 1.2 ± 0.22 1.30 ± 0.16	1.57 ± 0.23 1.08 ± 0.28 0.97 ± 0.19 1.51 ± 0.18^*^	1.09 ± 0.15 0.78 ± 0.18 1.35 ± 0.25 1.11 ± 0.18	1.15 ± 0.1 1.14 ± 0.17 1.18 ± 0.26 1.14 ± 0.1
**MAGAZINE LATENCY (s)**						
2 s before cue 1 s during cue First 3 s of the trial 5 s before cue	2.02 ± 0.4 1.68 ± 0.31 1.27 ± 0.22 2.05 ± 0.22	2.07 ± 0.35 1.94 ± 0.41 1.25 ± 0.2 1.94 ± 0.28	1.98 ± 0.14 1.79 ± 0.13 1.91 ± 0.22 2.63 ± 0.57	1.98 ± 0.12 1.81 ± 0.16 2.16 ± 0.29 2.05 ± 0.29	1.85 ± 0.16 2.37 ± 0.38 2.58 ± 0.58 2.03 ± 0.17	1.92 ± 0.25 2.46 ± 0.55 2.12 ± 0.45 1.87 ± 0.18
**PERSEVERATIVE RESPONSES ON TARGET (%)**						
2 s before cue 1 s during cue First 3 s of the trial 5 s before cue	0.07 ± 0.02 0.01 ± 0.01 0.06 ± 0.01 0.08 ± 0.03	0.05 ± 0.01 0.04 ± 0.04 0.02 ± 0.01 0.01 ± 0.01	0.05 ± 0.02 0.04 ± 0.02 0.03 ± 0.01 0.05 ± 0.02	0.07 ± 0.02 0.05 ± 0.03 0.05 ± 0.02 0.06 ± 0.02	0.1 ± 0.03 0.06 ± 0.02 0.02 ± 0.004 0.07 ± 0.01	0.07 ± 0.02 0.08 ± 0.04 0.05 ± 0.02 0.04 ± 0.01
**PERSEVERATIVE RESP OFF TARGET (%)**						
2 s before cue 1 s during cue First 3 s of the trial 5 s before cue	0.02 ± 0.01 0.08 ± 0.06 0.02 ± 0.01 0.08 ± 0.04	0.03 ± 0.02 0.08 ± 0.07 0.02 ± 0.01 0.04 ± 0.03	0.03 ± 0.01 0.004 ± 0.004 0.01 ± 0.01 0.01 ± 0.01	0.02 ± 0.01 0.02 ± 0.02 0.03 ± 0.01 0.02 ± 0.01	0.01 ± 0.01 0.03 ± 0.02 0.04 ± 0.02 0.04 ± 0.02	0.004 ± 0.004 0.004 ± 0.004 0.03 ± 0.01 0.01 ± 0.01

*Data are expressed as mean ± S.E.M. and asterisks represent significant differences between the light OFF vs. light ON condition in the same protocol*.

**Figure 5:**
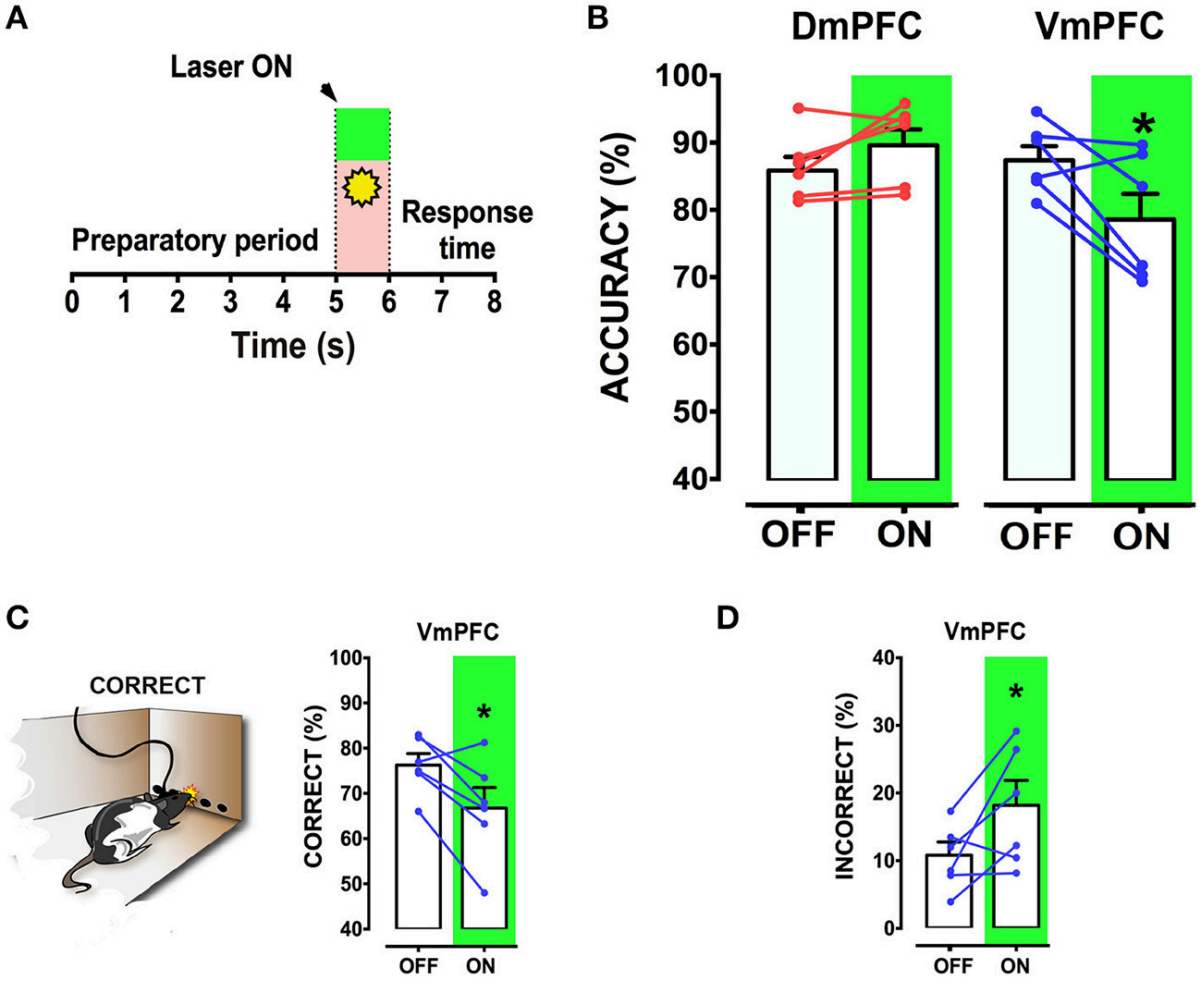
**VmPFC inhibition affects sustained attentional state during cue presentation: (A)** Graphical representation of the protocol used to optically inhibit mPFC neurons during cue presentation (CTRL: *n* = 5; DmPFC: *n* = 6; VmPFC: *n* = 6). **(B)** Accuracy of performance in DmPFC and VmPFC injected animals in light ON and light OFF trials. **(C,D)** Graphs showing the effect of the VmPFC inactivation on percent of correct and incorrect responses. Bar graphs are expressed as mean ± S.E.M.; lines report the performance per subject in the 2 different light conditions (ON vs. OFF). Asterisks indicate the result of the *post-hoc* multiple comparison Sidak's test. ^*^p < 0.05.

We next tested whether pyramidal neuron activity of the VmPFC or DmPFC is necessary during cue presentation for a proper sustained attentional state. Inhibition of VmPFC pyramidal neurons during cue presentation resulted in a reduction of the accuracy of responding [two-way repeated measures ANOVA: effect of light x virus interaction: *F*_(2, 14)_ = 4.393; *p* = 0.033; effect of virus: *F*_(2, 14)_ = 1.864; *p* = 0.192; effect of light: *F*_(1, 14)_ = 6.273; *p* = 0.025; Sidak's multiple comparison test OFF vs. ON: CTRL: *p* = 0.270; DmPFC: *p* = 0.826; VmPFC: *p* = 0.014; Figures 5A,B]. This effect was due to an increase of incorrect responses and a decrease in correct responses [two-way repeated measures ANOVA: effect of interaction light x virus correct: *F*_(2, 14)_ = 5.535; *p* = 0.017; Sidak's multiple comparison test OFF vs. ON: CTRL: *p* = 0.494; DmPFC: *p* = 0.524; VmPFC: *p* = 0.013; incorrect: effect of interaction light x virus: *F*_(2, 14)_ = 3.809; *p* = 0.048; Sidak's multiple comparison test OFF vs. ON: CTRL: *p* = 0.304; DmPFC: *p* = 0.714; VmPFC: *p* = 0.044; Figures 5C,D]. Also in this case, inhibition of DmPFC pyramidal neurons during cue presentation did not affect any parameter of performance (Figure 5B, Table 1). Thus, pyramidal neuron activity in the VmPFC is required during the preparatory phase, 2 s before cue presentation as well as during cue presentation itself, when rats are requested to prepare cue detection and to translate this into an instrumental response.

### Sustained inhibition of mPFC during a preparatory sustained attentional state

**Figure 6:**
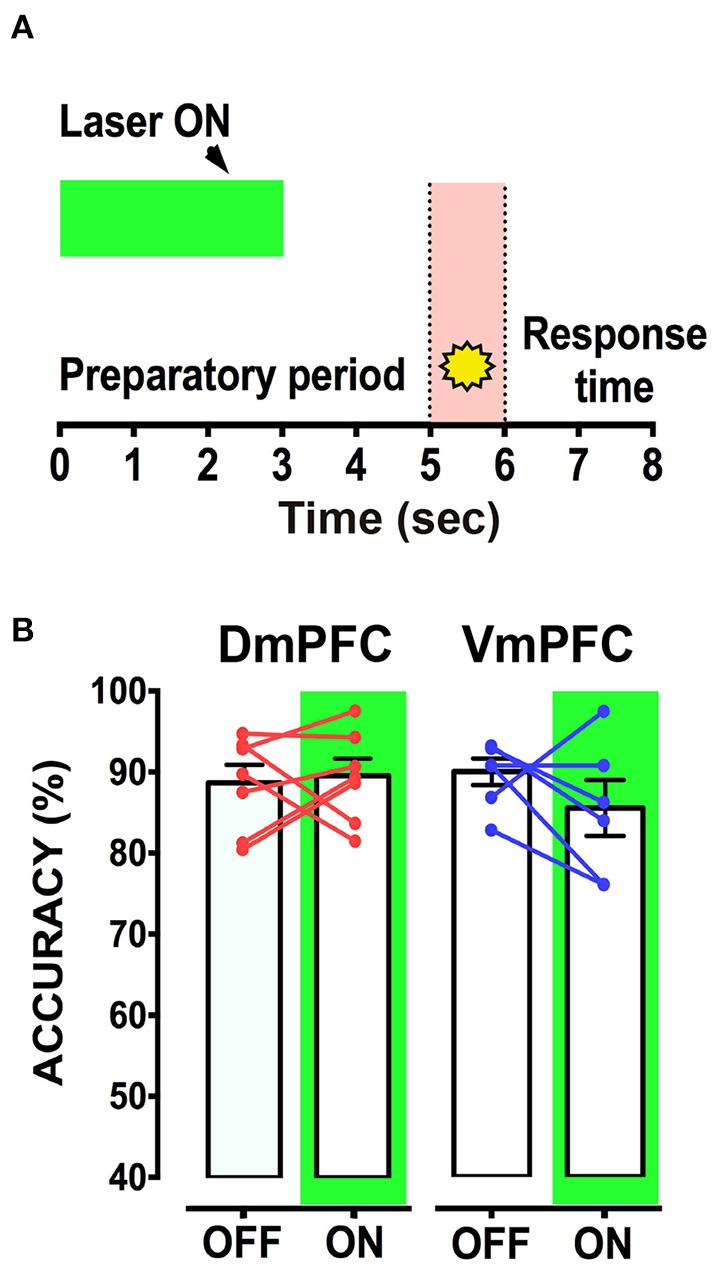
**mPFC inhibition during the first 3 s from trial onset does not affect sustained attentional state. (A)** Schematic representation of the protocol used to inhibit either DmPFC or VmPFC pyramidal cells in the first 3 s of the trial. (CTRL: *n* = 4; DmPFC: *n* = 7; VmPFC: *n* = 6); **(B)** Performance is not affected by the optogenetic manipulation of the mPFC in either Dm or VmPFC rats during the first 3 s of the trial, suggesting that optical inhibition in this epoch does not suffice to influence sustained attentional state.

**Figure 7:**
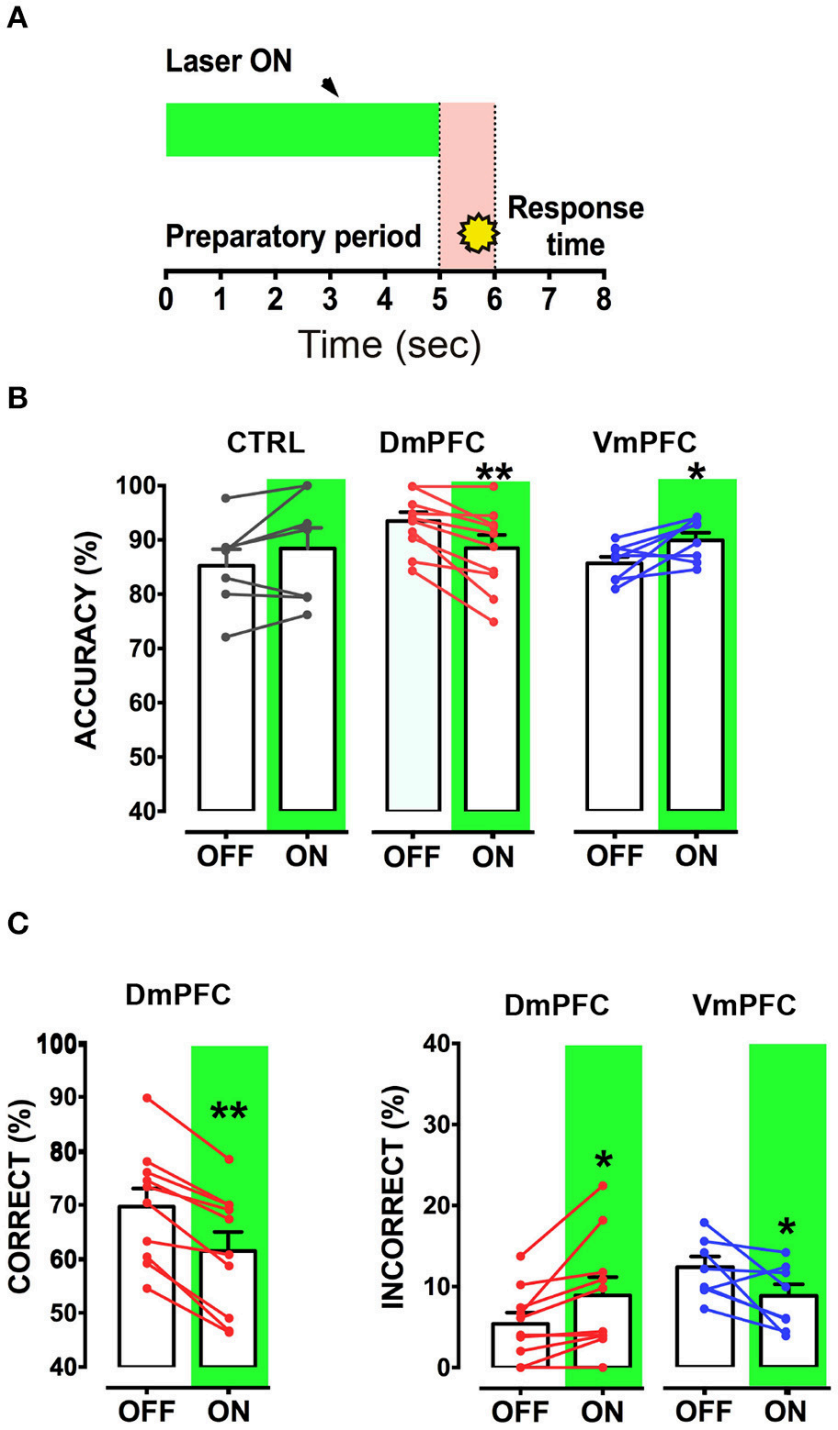
**Inhibition of DmPFC during the entire preparatory period reduces sustained attentional state. (A)** Graphical representation of the light protocol used, indicating that the laser was ON for half of the trials for 5 s before cue presentation. **(B)** Accuracy of performance in controls, DmPFC (*n* = 10), and VmPFC (*n* = 8) injected animals in light ON and light OFF trials. **(C)** Percentage of correct responses and incorrect responses that were significantly altered in the light ON condition. ^*^p < 0.05, ^**^p < 0.01.

Is the DmPFC causally involved in a sustained attentional state at these second time scales (Chudasama et al., 2003; Dalley et al., 2004; Totah et al., 2009)? To test whether activity of the VmPFC or DmPFC is required earlier in the task to guide a sustained attentional state, we inhibited pyramidal neurons in either the dorsal or the ventral mPFC for 3 s starting 5 s before cue presentation during the early phases of the preparatory sustained attentional state (Figure 6A). Optogenetic inhibition of VmPFC or DmPFC pyramidal neurons during this period did not affect any of the behavioral parameters in the task [two-way repeated measures ANOVA; effect of light x virus interaction: *F*_(2, 14)_ = 0.827; *p* = 0.457; effect of virus: *F*_(2, 14)_ = 0.514; *p* = 0.609; effect of light: *F*_(1, 14)_ = 1.238; *p* = 0.285, Figure 6B, Table 1]. In contrast, a sustained inhibition of the DmPFC for 5 s during the entire preparatory sustained attentional state (Figure 7A) did significantly affect the rodent accuracy of responding in the 5-CSRTT [two-way repeated measures ANOVA: effect of light x virus interaction *F*_(2, 22)_ = 11.760; *p* = 0.0003; effect of virus: *F*_(2, 22)_ = 0.849; *p* = 0.441; effect of light: *F*_(1, 22)_ = 0.856; *p* = 0.365; Sidak's multiple comparison test OFF vs. ON: CTRL: *p* = 0.194; DmPFC: *p* = 0.005; Figure 7B]. This effect was explained by a reduction in the percentage of correct responses, as well as an increase in the percentage of incorrect responses [two-way repeated measures ANOVA correct: effect of interaction *F*_(2, 22)_ = 14.790; *p* = 0.0001; Sidak's multiple comparison test OFF vs. ON: CTRL: *p* = 0.991; DmPFC: *p* = 0.0001; incorrect: *F*_(2, 22)_ = 9.199; *p* = 0.001; Sidak's multiple comparison test OFF vs. ON: CTRL: *p* = 0.268; DmPFC: *p* = 0.021; Figure 7C]. In addition, the response latencies for incorrect responses was significantly longer during ON trials, when compared to OFF trials (OFF vs. ON = 1.30 ± 0.16 s vs. 1.51 ± 0.18 s; paired *t*-test: *p* = 0.021) suggesting that prolonged inhibition of the DmPFC may interfere with responding to a cue.

Optical inhibition of the VmPFC during the entire 5 s of preparatory phase did not reduce control over a sustained attentional state, but to our surprise, slightly improved accurate responding, by decreasing the percentage of incorrect responses (Sidak's multiple comparison test OFF vs. ON accuracy: *p* = 0.037; % incorrect: *p* = 0.045; Figures 7B,C) while not affecting reaction latencies for both correct and incorrect responses (Correct response latency, OFF vs. ON: 0.62 ± 0.04 vs. 0.61 ± 0.04; paired *t*-test: *p* = 0.749; incorrect response latency, OFF vs. ON: 1.11 ± 0.18 vs. 1.14 ± 0.10; paired *t*-test: *p* = 0.863). Nevertheless, taken together, these results show that the requirements for neuronal activity in the DmPFC and VmPFC during a sustained attentional state are temporally dissociated.

## Discussion

In this study we found that pyramidal neurons in the DmPFC and VmPFC require distinct temporal activation profiles during a preparatory sustained attentional state. In particular, we found that the VmPFC plays an important role in the seconds that immediately precede and coincide with cue presentation. Transient inhibition of VmPFC pyramidal neurons during these seconds impairs visuospatial sustained attentional states as measured in the 5-CSRTT task and affects various parameters, including premature responses. In contrast, the visuospatial sustained attentional state is less sensitive to short inactivation of the DmPFC. Only when the DmPFC is inhibited for the entire preparatory phase before stimulus presentation and cue detection, a reduction in the sustained attentional state was observed. Since response latencies and errors of omission were not altered by optogenetic silencing, the observed findings were not secondary to changes in motor performance.

Even though a functional distinct role of different mPFC areas in cognitive functions has been previously shown, most of this evidence was obtained using tools that affect mPFC function on time scales far beyond the time scale for attentional processing (Passetti et al., 2002; Chudasama et al., 2003; Heidbreder and Groenewegen, 2003; Cassaday et al., 2014). As a result, a causal understanding of the temporal requirements of ventral and dorsal mPFC pyramidal neuron activity during different phases of attentional processing was lacking. In addition, due to the relatively low selectivity of these tools, previous studies have inactivated large portions of mPFC tissue hampering the understanding of the role of subregions in cognitive processes.

In fact, it is well known that the distribution of pyramidal neurons in the mPFC, as in the rest of the cortex, follows a laminar organization where different layers receive and send projections to different cortical and subcortical structures (Heidbreder and Groenewegen, 2003). For example while superficial layers of the mPFC (layer I and II/III) receive afferent projections from limbic and other cortical regions (Romanski et al., 1999), organize granular cortico-cortical communication (Douglas and Martin, 2004), and send compact projections to subcortical regions involved in impulse control (Hayton et al., 2010; Totah et al., 2013), deep layers (V and VI) might represent a crucial pathway for complex cognitive functions due to the relations with the mediodorsal thalamus (Gabbott et al., 2005; Kassam et al., 2008; Proulx et al., 2014) and due to their ability to integrate highly processed information from cortico-cortical and thalamic projecting neurons (Thomson, 2010; Proulx et al., 2014). In our study, we only inhibited the deep layers of the mPFC thereby sparing layer II/III pyramidal cells to provide further insights into activity of subclasses of cells within different mPFC subregions.

Optogenetic inhibition of the VmPFC in the seconds that precede cue presentation, as well as during cue presentation, revealed the driving role of this region in a sustained attentional state when a cue detection is required to produce an adaptive response. This provides additional evidence to support previous findings over the role of the prelimbic and infralimbic cortices in preparatory activity (Niki and Watanabe, 1979; Pragay et al., 1987; Totah et al., 2009).

In line with previous studies that induced prolonged inactivation of more ventral subcompartments of the mPFC by lesions or pharmacological inhibition (Passetti et al., 2002), we observed that transient and reversible optical inhibition of short epochs and during cue presentation resulted in a reduced suppression of undesired responses, i.e., increase in incorrect responding and increase in premature responding. Other studies have also shown that selective lesions of the PL/IL mantle, sparing ACg, are able to impair the preparatory processes in the condition movements triggered by the stimulus, affecting both the rate of correct responses and premature responses in a reaction time task (Risterucci et al., 2003), suggesting that VmPFC inhibition might also influence the instrumental response *per se*. Interestingly, we observed that the effect on undesired responses was primarily present when the manipulation immediately preceded stimulus presentation, and not observed when inhibitions were prolonged during the whole preparatory period, suggesting that pyramidal neuron-dependent withholding of non-desired responses might be a process that occurs late in the inter-trial interval. This is also in line with studies performed in the rodent PFC during visual and cross-modal attention tasks and auditory stimulus selection task that showed that this region might enhance neural representation of the target stimulus suppressing representation of other distractor stimuli (Miller and Cohen, 2001; Moore et al., 2003; Rodgers and Deweese, 2014; Wimmer et al., 2015). In particular, optogenetic perturbation of the PFC in mice performing a visual/auditory cognitive task reported impairment in the ability to select between conflicting sensory cues (Wimmer et al., 2015). As a consequence, it is then possible that our findings in the VmPFC might also be due to alterations in top-down control of a sustained attentional state that this subregion might exert on sensory regions before stimulus presentation.

We found that only short lapses of inhibition of ventromedial subregions affect performance in the 5-CSRTT. This may be explained by the fact that PL/IL have been regarded as pivotal players in representing the association between cue and response (Totah et al., 2009) and that IL cortex has been shown to be crucial in the modulation of habitual behaviors (Killcross and Coutureau, 2003; Smith et al., 2012). Thus, inhibition of the VmPFC in the seconds around stimulus presentation may primarily affect the planning of entering the illuminated port, also impairing the pattern of habitual responses which may be present in well-trained rodents (Totah et al., 2009), leading to more inappropriate response (e.g., too early as in the case of premature responses, or in a poorly adequate manner as in the case of incorrect nose-pokes).

It was previously found that rats with vast lesions of the PL/IL cortices or pharmacological inhibition of the mPFC showed increases in perseverative responses (Chudasama and Muir, 2001; Passetti et al., 2002; Murphy et al., 2012; Feja and Koch, 2014). We did not observe an increase in perseverative responding in our study, which may be explained by various reasons. First, the time-scale of our inhibition protocols was much smaller than the time scales from hours to week achieved with lesions or pharmacological agents. To increase perseveration may require longer mPFC inhibition for a behaviorally manifestation thereof. Second, since in our experiments opsins were expressed in the deep layers of the mPFC, it is possible that cognitive modules that suppress perseveration reside in upper layers rather than deeper layers of the mPFC. This is in line with evidence on a compact layer II/III projection to impulse-related subcortical regions, such as the core of the nucleus accumbens Pyramidal neurons in deep layers have been reported to exert a pivotal function in modulating (Hayton et al., 2010; Totah et al., 2013). Therefore, since we did not inhibit layers II/III of the VmPFC, this might explain the difference in findings on perseverative responding. Finally, the earlier studies inactivated the PL and IL cortices in their entirety, whereas in our study only the ventral part of the PL cortex and the dorsal part of the IL cortex were affected by optical manipulation. As a consequence, our protocols of inhibition may not have been targeted to a sufficiently large area to exert a sustained effect on perseveration in our animals. Future studies will have to clarify the specific temporal requirements and exact mPFC regions that control impulsive and compulsive responses.

Deactivation of the DmPFC during the entire preparatory period reduced the sustained attentional state, whereas transient inhibition of the DmPFC for only 3 s at the start of the preparatory phase or immediately preceding cue presentation and during cue presentation, had no effect on the sustained attentional state. This suggests that the ACg and dorsal PL have an active role in preparatory processing, but the timing of DmPFC activity is not strictly time-locked to the cue. As long as the DmPFC was not inhibited during the entire preparatory phase, 5-CSRTT performance was unaffected. Neuronal activity in the ACg is increased during a preparatory sustained attentional state (Totah et al., 2009), and relatively long-lasting chemogenetic inhibition of this area reduced attention-related performance in mice (Koike et al., 2015). The DmPFC is interconnected with a number of cortical and subcortical regions among which the sensorimotor areas (Sesack et al., 1989) and the visual cortex (Sesack et al., 1989; Zhang et al., 2014; Zingg et al., 2014; Koike et al., 2015) and recent electrophysiological observations have shown that afferents from the mediodorsal thalamus promote feed-forward inhibition of ACg pyramidal cells via recruitment of parvalbumin-containing interneurons modulating the network activity that is crucial to maintain adaptive behaviors (Delevich et al., 2015). Therefore, it is likely that long-lasting inhibition might have hampered the communication between DmPFC and other brain regions that hold and manipulate the sensory representation of the imminent cue, and/or might have dysregulated the delicate excitation/inhibition balance that is maintained functional by inhibitory parvalbumin-positive interneurons. This may suggest that the DmPFC plays a role in cognitive and sensory flexible representation of the rule to respond into the illuminated port.

Other studies have indeed shown that the ACg/DmPFC is involved in representing the task-rules in a set-shifting performance task (Park et al., 2016), may be sequencing temporally ordered behaviors in a go/no-go task (Delatour and Gisquet-Verrier, 2001), and is able to maintain the task-rule across delay periods before a response in a win-shift radial arm maze task (Gisquet-Verrier and Delatour, 2006).

Notably, the mPFC is also involved in a number of other behavioral functions that may be interrelated with attentional processing. For example, it has been shown that PL and IL cortices exert opposing roles in the expression and extinction of fear responses (Gourley and Taylor, 2016) and that silencing of IL projections to the basomedial amygdala causes increase in anxiety (Adhikari et al., 2015). Moreover, whereas the IL seems more crucial for habitual behaviors, the PL compartment might be more influential in developing goal-directed behaviors (Gourley and Taylor, 2016). Future work is warranted to unravel as to what extent these other behavioral functions relate to the current findings.

Surprisingly, we also observed that sustained inhibition of the VmPFC during the entire preparatory phase of a sustained attentional state slightly improved accuracy of responding, in contrast to the short inhibition protocols. It is at this point not clear how the 5 s inhibition of deep layers of the VmPFC led to improvement of performance. Possibly, the inhibition of the deep layers was compensated for by activation of other PFC regions, since PFC subregions are anatomically and functionally interconnected (Gabbott et al., 2005; van Aerde et al., 2008; Totah et al., 2009; Murphy et al., 2012; Pezze et al., 2014). Alternatively, the 5-s long inhibition of the VmPFC may have resulted in circuit re-modulation and change in functionality. Recordings of unit activity within the medial PFC during a visuospatial task showed that neurons can change their activity in opposite directions, either increasing or decreasing their activity (Totah et al., 2009, 2013). Optogenetic inhibition of pyramidal neuron activity as we did here may favor neurons that reduce their activity during the preparatory period of a sustained attentional state. How this translates into behavioral performance is not understood.

Our findings reveal that pyramidal neurons in the VmPFC and DmPFC require distinct temporal activation profiles during a sustained attentional state. Albeit effect sizes on performance were in the order of 5–10% (from baseline levels of approximately 85%) and as such may seem modest, they were very consistent across rats. Given the strong connectivity that the mPFC has with other cortical and subcortical structures, and the relative quick optical manipulations we used it is also possible that changes we observed in some of our parameters may result at least in part from propagated network activity in afferent/efferent structures rather than a direct engagement of pyramidal cells.

**Figure 8:**
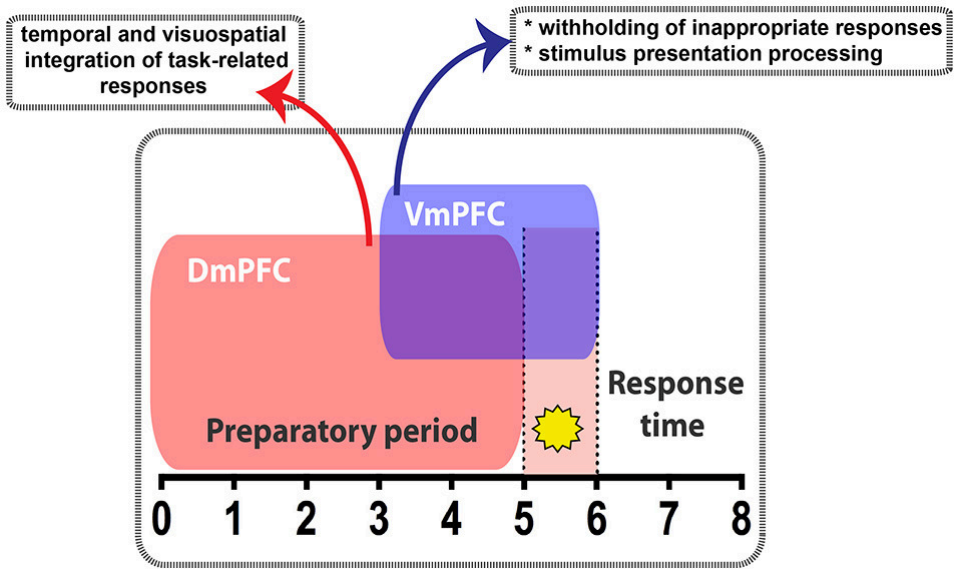
**Diagram summarizing the main findings of this study**. During the 5CSRTT performance, temporally segregated manipulation of pyramidal neuron activity in either the Dm- or the VmPFC exert differential effect. VmPFC activity is necessary in the seconds that precede and coincide with the stimulus presentation (yellow star) where it might play a role in withholding the unwanted responses and process the information of the stimulus. DmPFC is required throughout the whole preparatory period to likely integrate the temporal and visuospatial aspect related to the task.

Activity in the VmPFC is strictly time-locked to cue onset and is required shortly before and during cue presentation, whereas activity of DmPFC is temporally more loosely associated with cue onset, but is required during the preparatory phase of sustained attentional states. Thus, our results show that a dissociable temporal recruitment of VmPFC and DmPFC in cognitive functions exists during sustained attentional states as measured by the 5-CSRTT. During the preparatory sustained attentional state, the VmPFC controls behavior by withholding inappropriate responses and by processing the imminent stimulus presentation (Passetti et al., 2002; Paine et al., 2011; Murphy et al., 2012), whereas the DmPFC may integrate temporal and visuospatial information (Sesack et al., 1989) to temporally organize task-related responding (e.g., rule to enter the illuminated port) (Figure 8). It is interesting to note that studies employing prefrontocortical electrophysiological recordings during selective attention tasks in macaque, and other non-human primates also underscored a functional dissociation between the activity of the ACg and the VmPFC. In this regard, it has been observed that while confined clusters of neurons in the macaque VmPFC transfer stimulus information values during task performance, ACg neurons predict the stimulus location to allow shifts in attentive state (Kaping et al., 2011). Moreover, whereas ventrolateral regions of the PFC might maintain internal stimulus representations, more dorsal PFC regions might manipulate this information for task-relevant aspects (Petrides, 2000).

To conclude, our interventions may reveal the timing requirements to modulate cortical and subcortical areas to set up control over attentional processing in the context of reward expectation (Gruber et al., 2009; Totah et al., 2009) and prepare the organism to integrate cognitive and sensory inputs to produce adaptive responses to achieve a goal.

## Author contributions

HM obtained funding for this study. HM, TP, and AL designed the study. KD provided viral tools. AL and OM performed surgeries, behavior, perfusions, and anatomy experiments. HT, BB, and SD assisted in the training, behavior and anatomy experiments. RD and CD provided analysis tools and MATLAB scripts. AL, HM, and TP analyzed the behavioral data. JO assisted with transcardial perfusions, and together with TH performed *ex vivo* electrophysiology experiments. JO, TH, and HM analyzed the electrophysiological data. AL, HM, and TP wrote the manuscript with input from all other authors.

## Funding

HM received funding for this work from the Netherlands Organization for Scientific Research (NWO; 917.76.360, 912.06.148, and a VICI grant), ERC StG “BrainSignals,” the Dutch Fund for Economic Structure Reinforcement (FES, 0908 “NeuroBasic PharmaPhenomics project”), EU 7th Framework Programme (HEALTH-F2-2009-242167 “SynSys” and agreement no. 604102 “Human Brain Project”).

### Conflict of interest statement

The authors declare that the research was conducted in the absence of any commercial or financial relationships that could be construed as a potential conflict of interest.
